# Assessment of Acute-Phase Protein Response Associated with the Different Pathological Forms of Bovine Paratuberculosis

**DOI:** 10.3390/ani10101925

**Published:** 2020-10-20

**Authors:** José Espinosa, Rubén de la Morena, Julio Benavides, Carlos García-Pariente, Miguel Fernández, Miguel Tesouro, Noive Arteche, Raquel Vallejo, M. Carmen Ferreras, Valentín Pérez

**Affiliations:** 1 Departamento de Sanidad Animal, Facultad de Veterinaria, Campus de Vegazana s/n, Universidad de León, 24071 León, Spain; rmorev00@estudiantes.unileon.es (R.d.l.M.); cgpariente@gmail.com (C.G.-P.); m.fernandez@unileon.es (M.F.); nartv@unileon.es (N.A.); rvalg@unileon.es (R.V.); mcfere@unileon.es (M.C.F.); vperp@unileon.es (V.P.); 2 Departamento de Sanidad Animal, Instituto de Ganadería de Montaña (CSIC-Universidad de León), Finca Marzanas, Grulleros, 24346 León, Spain; julio.benavides@csic.es; 3 Departamento de Medicina, Cirugía y Anatomía Veterinaria, Facultad de Veterinaria, Campus de Vegazana, 24071 León, Spain; matesd@unileon.es

**Keywords:** bovine, haptoglobin, serum amyloid A, *Mycobacterium avium* subsp. *paratuberculosis*, types of lesion, serum samples

## Abstract

**Simple Summary:**

Paratuberculosis (PTB) is a chronic debilitating disease caused by *Mycobacterium avium* subspecies *paratuberculosis* (Map) that affects ruminants worldwide. Many aspects related to the pathogenesis of this disease are still unknown, including the inflammatory acute-phase response developed during the course of the infection. To clarify this, serum levels of haptoglobin and serum amyloid A, two positive acute-phase proteins, were evaluated in a total of 190 cows, among which 59 were healthy control animals and 131 cows were diagnosed post-mortem with different types of lesion associated with Map-infection. The results reflect a significant increase of these proteins’ levels in the infected animals and, more specifically, in those animals with types of lesion characterized by a low bacterial load and with predominance of a cell-mediated immune response. This suggests that these molecules would play a certain role in the pathogenesis of the PTB and a possible utility as biomarkers of different stages of the disease.

**Abstract:**

In this study, the concentrations of two acute-phase proteins (APPs), haptoglobin (Hp) and serum amyloid A (SAA), were quantitatively assessed in serum samples from cattle naturally infected with paratuberculosis (PTB). APP profiles were compared across 190 animals classified according to the different pathological forms associated with infection: uninfected (*n* = 59), with focal lesions (*n* = 73), multifocal lesions (*n* = 19), and diffuse paucibacillary (*n* = 11) and diffuse multibacillary lesions (*n* = 28). Our results showed a significant increase in both APPs in infected animals compared to the control group, with differences depending on the type of lesion. Hp and SAA levels were increased significantly in all infected animals, except in cows with diffuse multibacillary lesions that showed similar values to non-infected animals. The expression pattern of both APPs was similar and negatively correlated with the antibody levels against PTB. These results indicate that the release of Hp and SAA is related to the presence of PTB lesions associated with a high cell-mediated immune response and a lower bacterial load, suggesting that the pro-inflammatory cytokines that are associated with these forms are the main stimulus for their synthesis. These molecules could show some potential to be used as putative biomarkers of PTB infection, particularly for the identification of subclinical animals showing pathological forms related to latency or resistance to the development of advanced lesions.

## 1. Introduction

Paratuberculosis (PTB) is a chronic debilitating disease characterized by a granulomatous enteritis and lymphadenitis that affects ruminants worldwide, caused by the facultative intracellular pathogen *Mycobacterium avium* subspecies *paratuberculosis* (Map) and responsible for high economic losses to domestic livestock [1]. Animals typically get exposed to Map early in life, mainly through the fecal–oral route. After infection, the clinical course of the disease may follow different paths. Some animals are resistant to infection [2,3], while others, when they fail to clear the initial infection, may show resilience to disease [4] and remain in a subclinical or asymptomatic stage for a long period of time, even their entire life, in which infection is controlled. In this sense, the formation of granulomas at the site of Map infection is critical. After several years, a certain number of these chronically infected animals can gradually develop clinical signs that are associated with a diffuse granulomatous enteritis, which widely affects several areas of the intestine with variations according to the main inflammatory cells present in the infiltrate and the amount of Map, so that two main forms are recognized: multibacillary and paucibacillary forms [5,6].The development of these different lesions is closely related to the immune response mounted by the host in each phase of the infection [7,8]. Specifically, a cellular Th1 type response with the production of specific cytokines such as IFN-γ, TNF-α, IL-1, IL-6, or IL-23 predominates in the early and subclinical or latent stages of Map infection, shifting to a humoral Th2 type with the predominance of cytokines such as IL-4 and IL-10 in the final clinical stages of the disease [9,10]. Some of these cytokines, such as TNF-α, IL-1, and IL-6, also stimulate the production of acute-phase proteins (APPs) [11,12].

The APPs are a group of serum proteins that are produced and released mainly by hepatocytes during an inflammatory challenge as part of the acute-phase response, although they are also released during the sub-acute and chronic phases of the inflammation [13,14]. In ruminants, haptoglobin (Hp) and serum amyloid A (SAA) are known to be the major positive acute-phase proteins increasing in relation to trauma, infection, stress, or neoplasia [15,16]. The circulating concentration of these APPs is related to the severity of the disorder and the extent of tissue damage in the affected animal, therefore giving not only diagnostic but even prognostic value [17]. In this sense, the influence of different inflammatory conditions on the concentrations of these proteins in domestic livestock has been documented, such as in various bacterial and metabolic disorders such as metritis [18], mastitis [19], caseous lymphadenitis [20], respiratory infections [21], or ruminal acidosis [22]; parasitic diseases such as psoroptic and sarcoptic mange [23,24];or viral conditions as Schmallenberg virus infection [25], bovine viral diarrhea [26], blue tongue disease [27], or foot-and-mouth disease [28], among others. 

In animal medicine, although the research carried out in this field is extensive, the number of studies that analyze the pattern of response of APPs in mycobacterial infections is very rare. Existing studies have been focused on tuberculous animals [29,30], and the present knowledge about acute-phase response during PTB infection is scarce. Therefore, the main objective of this study was to analyze serum concentrations of Hp and SAA in non-infected and naturally Map-infected cows with the following purposes: (1) to determine the variation in the levels of these proteins according to the status or phase of PTB infection, assessed by pathological methods; (2) to analyze the possible relationship between Hp and SAA serum levels with the different types of lesions associated with bovine PTB; (3) to assess the possible correlation between Hp and SAA serum values, as well as with the antibody levels against Map; and (4) to discuss the possible role of these molecules as biomarkers of the disease and as a diagnostic tool.

## 2. Materials and Methods 

### 2.1. Experimental Design

Intestinal and serum samples from 190 adult Holstein Friesian cows (ranging from three to six years of age) were analyzed in this study. The animals came from two commercial herds affected by PTB, located in the “Castilla y León” region (Spain) and had been culled according to the standard methods in the current legislation, in an authorized slaughterhouse. The entire intestine and the associated lymph nodes were immediately collected and transferred to the Department of Animal Health of the Faculty of Veterinary Science (University of León, León, Spain). Parts of samples were stored at −20 °C for other studies and the rest of the tissues were fixed in 10% buffered formalin. Before slaughter, blood samples were collected from each animal by jugular venipuncture into 10 mL evacuated tubes (Vacutainer^®^, Becton Dickinson, Plymouth, UK) without anticoagulant. Blood samples were allowed to clot at room temperature. Then, the serum was obtained by centrifugation at 4750× *g* for 10 min and stored at −20 °C until analysis. In all animals included in the study, no gross or histologic lesions compatible with other inflammatory, infectious, or parasitic processes were identified during post-mortem inspection. 

### 2.2. Diagnosis of Paratuberculosis Infection

#### 2.2.1. Humoral Immune Response Determination by Indirect ELISA (Ab ELISA)

Serological testing for production of antibodies (Ab) against Map was determined in all animals of the study using an indirect ELISA validated for domestic cattle, ID Screen^®^ Paratuberculosis Indirect (IDVet, Grabels, France). This ELISA has a reported sensitivity of 41.5% and specificity of 99.42% [31,32]. The test is a *Mycobacterium phlei* absorbed ELISA detecting anti-Map immunoglobulin G. The technique was performed according to the manufacturer’s instructions for bovine samples. The absorbance values were measured spectrophotometrically at 450 nm using an ELX800 ELISA reader (Bio-Tek Instruments, Winooski, VT, USA). The results were expressed as a quotient between the mean OD of each sample serum and the mean OD of the positive control serum in each plate.

#### 2.2.2. Tissue Samples and Classification of PTB Type of Lesion

Selected intestinal samples were macroscopically evaluated and sections were collected for histopathological analysis. Specifically, samples from ileum, jejunum (with and without Peyer´s patches), ileocaecal valve and ileocaecal, and jejunal lymph nodes were taken from each animal. Samples fixed in 10% buffered formalin were processed in a conventional way through a graded alcohol series and xylol, before being embedded in paraffin wax. Tissue sections 2.5 μm thick were obtained from each sample and stained with Harris’s hematoxylin and eosin (H&E) and Ziehl–Neelsen method for acid-fast bacilli (AFB) identification. No lesions consistent with Map infection were observed in 59 cattle, whereas granulomatous lesions were detected in different samples from the intestine and lymph nodes in the remaining animals. Lesions were classified into 4 categories, namely focal, multifocal, diffuse paucibacillary, and diffuse multibacillary, according to the presence and location of granulomas in the different intestinal compartments, cell types present in the inflammatory infiltrate, and the amount of AFB [5]. Each animal was classified depending on the most severe lesion found in the examined samples, bearing in mind that the type of lesion could vary among the tissue samples obtained from the same individual. Focal lesions were characterized by the presence of small, well-demarcated granulomas composed of 5–40 epithelioid cells located exclusively in the interfollicular areas of the intestinal lymphoid tissue. AFB were absent or detected in very low amounts in the cytoplasm of the macrophages. This type of lesion was present in 73 Map-infected cows. Multifocal lesions were similar to the focal forms, except for the presence of small granulomas also in areas of the lamina propria adjacent or not to the lymphoid tissue, without altering the histological structure of the intestine. This type of lesion was observed in 19 animals. Diffuse lesions were characterized by widespread granulomatous enteritis that significantly modified the normal intestinal architecture. Lesion was present in areas of the intestine (ileum and jejunum) both with and without lymphoid tissue. Large numbers of macrophages and occasional Langhans-type giant cells appeared in the interfollicular areas, infiltrating the lymphoid follicles. The lamina propria was markedly thickened and distended due to the presence of a severe granulomatous infiltrate. According to the cellular types and number of AFB, 2 types of diffuse forms were considered as follows: diffuse paucibacillary lesions, seen in 11 Map-infected cows, where the infiltrate was composed mainly of lymphocytes, with some macrophages and giant cells, and occasional AFB located in them; and diffuse multibacillary lesions, identified in another 28 Map-infected cows, characterized by the predominance of macrophages morphologically consistent with epithelioid cells and some multinucleated giant cells, harboring large numbers of AFB, and low numbers of lymphocytes.

#### 2.2.3. Nested PCR

Sections from the same paraffin blocks that were employed for lesion classification were used for molecular detection of Map DNA using the nested PCR method. DNA was isolated using SpeedTools Tissue DNA extraction kit according to the manufacturer’s instructions (Biotools^®^ B&M Labs., Madrid, Spain). The nested PCR was carried out as previously described [33] using primers to detect the presence of Map-specific IS*900* DNA. All samples were analyzed in duplicate and considered positive for PCR reaction when Map DNA was detected in at least one of them.

Animals were classified as positive to PTB infection when histological lesions, i.e., focal/multifocal or diffuse granulomatous enteritis, were observed and Map DNA was identified on PCR amplification. In all animals with granulomatous lesions employed in the study, the presence of Map infection was confirmed by PCR technique. All animals without microscopic lesions showed a negative PCR result.

### 2.3. Serum Acute-Phase Protein(APP) Determination

Serum Hp concentrations were quantified using a colorimetric Phase^™^ Haptoglobin Assay kit (Tridelta Development Limited, Maynooth, Ireland), based on peroxidase activity of the haptoglobin–hemoglobin complex at low pH, according to the manufacturer’s instructions. SAA concentrations were determined using a commercial Phase^™^ Range Multispecies SAA ELISA kit (Tridelta Development Limited, Maynooth, Ireland). In this case, sera were initially diluted 1:100 in buffered saline diluent. All the samples used for Hp and SAA determinations were run in duplicate, and the mean of each duplicate was used to determine final concentration. Samples with a signal greater than the highest standard were diluted and re-run until all the signals fell within the linear part of the standard curve. The final absorbances of both techniques were immediately read at 630 nm for Hp, and at 450 nm using 630 nm wavelengths as a reference for SAA, all of them in an ELX 800 ELISA Microplate Spectrophotometer (Bio-Tek Instruments, Inc., Winooski, VT, USA). The final values were expressed in mg/mL for Hp and μg/mL for SAA concentrations.

### 2.4. Statistical Analysis

The results of the ELISA index values (serum Ab) as well as the Hp and SAA concentrations according to the different types of lesions analyzed, were expressed as mean, standard deviations, and range (minimum and maximum), and calculated using conventional descriptive statistical procedures. The Kolmogorov–Smirnov test with the “nortest” package was used to assess the data normality. Since none of the variables analyzed fitted a normal distribution, non-parametric tests were used to make comparisons between groups. Specifically, to compare the serum Ab levels, represented by the ELISA index, and the different APP concentrations studied, either between the different infection status (uninfected/infected) or the different types of lesion (non-lesion, focal, multifocal, diffuse paucibacillary, and diffuse multibacillary), the Mann–Whitney *U* and Kruskal–Wallis tests were used, respectively. In a second step, in order to know between which pair of groups the differences were found, a post-hoc analysis (pairwise Wilcoxon rank sum test) with Bonferroni correction was performed for the level of significance [34]. Finally, the Spearman’s rank correlation test was applied for the calculation of the *rho* coefficients among the different variables analyzed. *p*-values < 0.05 were considered to be statistically significant. 

All statistical analyses were performed with the R software version 3.5.3 (Vienna, Austria) [35].

## 3. Results

The ELISA index varied significantly (*W* = 48.18, *p* = 0.003) between non-infected (1.33 ± 0.12) and Map-infected (1.87 ± 0.15) animals, regardless of the type of lesion. Similarly, these values varied significantly (χ^2^ = 41.66, *df* = 4, *p* < 0.001) when the different types of lesion were analyzed. As it can be seen in Table 1, the Ab levels against PTB increased significantly, compared to the control or non-lesion group, in animals with multifocal forms (*p* = 0.023), diffuse paucibacillary (*p* = 0.008), and diffuse multibacillary lesions (*p* < 0.001), the latter showing the highest levels, being 2.33 times higher than non-infected animals. No significant differences were observed between cows with focal forms and those without lesions (*p* = 0.262). Finally, there were differences in the production of Ab between both diffuse forms (*p* < 0.01).

The Hp concentrations also varied significantly (*W* = 51.38, *p* < 0.001) between control (0.33 ± 0.05) and infected (0.72 ± 0.08) animals. This variation was also evident when the different types of lesion were considered (χ^2^ = 25.57, *df* = 4, *p* < 0.001). All Map-infected animals showed higher levels of Hp than the non-lesion group. However, only in the cows with focal (*p* < 0.01), multifocal (*p* = 0.009), and diffuse paucibacillary (*p* < 0.001) forms were these differences statistically significant compared to animals without lesion. The highest Hp values were observed in those cows with diffuse paucibacillary forms, with values 3.93 times higher than the control group, followed by those with focal and multifocal lesions, whereas among animals with lesions, the lowest levels were observed in the multibacillary diffuse forms, showing values 3.85 times lower than diffuse paucibacillary forms, approaching the values shown by the non-infected animals (*p* = 0.872) (see Table 1).

Similarly to Hp concentrations, serum SAA levels varied significantly (*W* = 42.11, *p* < 0.001) between control (6.91 ± 0.56) and Map-infected (12.54 ± 0.76) cows. When considering the different types of lesion, SAA concentrations were also modified (χ^2^ = 40.71, *df* = 4, *p* < 0.001). Specifically, only animals that developed focal (*p* < 0.01), multifocal (*p* < 0.01), and diffuse paucibacillary (*p* < 0.001) lesions showed significantly higher SAA levels than the control or non-lesion group. The highest levels, as observed in the case of Hp, were identified in cows with diffuse paucibacillary forms, which were 2.86 times higher than non-infected animals, followed by those with multifocal and focal forms. In contrast, in animals with diffuse multibacillary lesions, the mean SAA concentrations were the lowest observed (3.07 times less than diffuse paucibacillary forms), even lower than those detected in cows without lesion, although in the latter case, this variation was not significant (*p* = 0.703).

Finally, when jointly analyzing the mean levels of the three parameters assessed (Ab levels, Hp and SAA concentrations), it was observed that the Ab levels against Map were negatively and significantly correlated with the Hp (r_s_ = −0.11, *p* = 0.025) and SAA (r_s_ = −0.90, *p* = 0.029) production. In contrast, the production of both APPs showed a similar production pattern, both being positively correlated (r_s_ = 0.21, *p* < 0.001).

## 4. Discussion

To the best of our knowledge, although there is a brief mention of the acute-phase response assessment in Map-infected sheep in a previous study [23], this is the first report that deeply analyzes the Hp and SAA serum values in cows infected with Map in relation to the different pathological forms associated with the disease, both from histological and serological perspectives. A positive association between circulating serum Hp and SAA levels in relation to Map-infection status was observed. This finding shows that an acute-phase response (in terms of Hp and SAA production) develops and is maintained in Map-infected cows over time, in spite of the chronic character of PTB. According to these results, both Hp and SAA could be classified as moderate APPs (threefold increase) [36] that play a role in the inflammatory process induced by Map infection.

However, although the levels of both proteins increased during infection, the pattern of response was different when analyzing the different clinical stages and pathological forms of PTB. In most of the conditions evaluated in ruminants, the increase in the APPs’ levels was proportional to the severity of the lesions and clinical signs [18,19,20,21,22,23,24,25,26,27,28]. In the case of mycobacterial diseases, studies carried out in tuberculous animals also showed increases in serum APPs according to the severity of the lesions present [29,30]. Similar results were observed in the case of human tuberculosis [37,38]. However, in PTB infection, this increase was not detected in all the cases with severe clinical signs and lesions. It occurred only in those showing diffuse paucibacillary forms, whereas in cows with diffuse multibacillary forms, also severe in terms of clinical signs and lesions, the levels were lower than those observed in the initial stages of the disease (focal and multifocal lesions), with values very similar to those of the non-infected animals. This change in the variation of the acute-phase response between the different pathological forms could be explained considering the predominant immune response in each of them. 

During PTB infection, the development of focal, multifocal, and diffuse paucibacillary lesions has been associated with an intense Th1 cell-mediated immune response with a predominance of pro-inflammatory cytokines such as TNF-α, IL-1, IL-6, and IL -23 [9,39,40], which, at the same time, are the main stimulus for APPs production at the hepatic level [41]. This is consistent with the fact that the highest Hp and SAA serum values have been observed in these three types of lesion. However, in those animals that develop diffuse multibacillary lesions, a Th2 humoral-type immune response predominates with the production of anti-inflammatory cytokines such as IL-4 or IL-10 [42,43]. In this case, this anti-inflammatory response would be responsible for the lack of hepatic stimulation during the acute-phase response and, therefore, the lowest levels of both proteins detected in cows with diffuse multibacillary lesions. These serum values, although from the pathological point of view they may differ from those observed in tuberculosis [30,37], they are similar if they are analyzed according to the type of immune response in each stage of the disease, since the most severe pathological forms of tuberculosis considered in the previous studies [29,30,37,38] are also associated with a cell-mediated immune response [44], similar to that observed in diffuse paucibacillary forms of PTB. In the case of human leprosy, another mycobacterial disease, those patients suffering lepromatous forms, with great similarities to the multibacillary diffuse type of PTB, showed a similar pattern of response, with low APPs production [45], as long as no other associated secondary disorders such as systemic amyloidosis were present [46].

Nonetheless, both Hp and SAA might play a functional role against disease. The mechanisms involved may be related to the functionality of both APPs, since they show bactericidal activity. The main function of Hp is to bind iron from free hemoglobin released by erythrocytes or iron-containing proteins resulting from tissue damage, limiting its availability for bacterial growth [12]. In turn, SAA has been shown to promote chemotactic recruitment of inflammatory cells to sites of lesion, increasing macrophage and neutrophil phagocytosis [47,48]. The fact that the levels of both APPs were higher in the focal, multifocal, and diffuse paucibacillary types of lesion, where the presence of AFB in the tissues is null or scarce, and lower in those lesions where there is a large number of bacilli (diffuse multibacillary forms) [5] supports this hypothesis, suggesting that both APPs may contribute to the limitation of Map multiplication in the tissues of Map-infected cows. However, additional studies would be needed to confirm this hypothesis.

In this study, a positive correlation was also observed between serum Hp and SAA values, with a similar increase in the different stages of PTB. This seems to indicate that both APPs would share the same production and release mechanisms probably initiated by the same stimuli. In contrast, serum levels of Hp and SAA showed a pattern of response opposite to the Ab serum production during PTB infection. This is consistent with the fact that the presence of pro-inflammatory cytokines, necessary for its production, reaches the highest levels in animals with focal, multifocal, and diffuse paucibacillary lesions, those in which there is a predominance of a cell-mediated immune response, and where the Ab production, which reaches its highest levels in diffuse multibacillary forms, is still very low [8,49].

APP responses in animals are highly unspecific and can be altered by multiple causes related or not to the primary infection. Despite this, in the case of PTB, the fact that the levels of both proteins are significantly increased in infected animals compared to healthy individuals, in which no other evident disorders were observed, raises the possibility of the use of these molecules as biomarkers of infection. In view of our results, these proteins could be used for the identification of Map-infected cows with pathological forms related to strong cell-mediated immune responses. Except for those having diffuse paucibacillary forms that are linked to evident clinical signs, they correspond to animals with focal or multifocal lesions, associated with stages of latency or resistance to the development of clinical disease [3,4]. However, due to their lack of specificity, we consider that these markers should be used in combination with diagnostic tests traditionally used to detect cell-mediated or antibody responses against Map infection.

## 5. Conclusions

This study analyzes for the first time the acute-phase response in the different stages of PTB, showing that increased levels of APPs in blood are present in animals showing chronic lesions. Moreover, the results of this study demonstrate that the APPs’ levels in bovine PTB vary according to the different pathological forms, linked to the presence of clinical signs. They are higher in those animals in which a cell-mediated immune response predominates. Therefore, we may conclude that APPs show some potential as a marker for the identification of subclinically Map-infected cows bearing pathological forms related to latency or resistance to the development of advanced clinical stages. Due to the challenges associated with their detection, the use of Hp and SAA as markers for a diagnostic approach should be complemented by other more specific diagnostic techniques. All these findings will contribute to further elucidating the pathogenesis of this disease, although future studies are needed to better understand the role of these proteins in the development of PTB.

## Figures and Tables

**Table 1:** Means ± standard deviations and range (minimum–maximum) of ELISA index values and haptoglobin (Hp) and serum amyloid A (SAA) concentrations in non-infected animals and *Mycobacterium avium* subspecies *paratuberculosis* (Map)-infected cows, according to the different types of lesion.

	Infection Status				
	Non-Infected Animals (*n* = 59)	Map-Infected Animals (*n* = 131)			
		Types of Lesion			
		Focal (*n* = 73)	Multifocal (*n* = 19)	Diffuse Paucibacillary (*n* = 11)	Diffuse Multibacillary (*n* = 28)
ELISA index	1.33 ± 1.22 ^a^ (0.063–4.02)	1.31 ± 1.17 ^a^ (0.073–4.16)	1.69 ± 1.43 ^b^ (0.25–3.93)	1.76 ± 1.56 ^b^ (0.096–3.94)	3.10 ± 1.02 ^c^ (0.72–3.28)
Hp (mg/mL)	0.33 ± 0.46 ^a^ (0.033–2.19)	0.80 ± 1.06 ^b^ (0.15–5.45)	0.65 ± 0.56 ^b^ (0.12–2.23)	1.30 ± 1.29 ^c^ (0.09–3.68)	0.35 ± 0.49 ^a^ (0.05–2.21)
SAA (μL/mL)	6.91 ± 3.98 ^a^ (1.99 ± 20.84)	13.10 ± 8.48 ^b^ (1.42–38.94)	13.56 ± 6.63 ^b^ (2.79–27.14)	19.82 ± 10.09 ^c^ (8.67–41.09)	6.44 ± 2.58 ^a^ (2.18–11.04)

Different superscripts within each row indicate statistically significant differences.
